# Health workers’ views on factors affecting caregiver engagement with bubble CPAP

**DOI:** 10.1186/s12887-020-02088-8

**Published:** 2020-04-23

**Authors:** Sangwani Salimu, Mai-Lei Woo Kinshella, Marianne Vidler, Mwai Banda, Laura Newberry, Queen Dube, Elizabeth M. Molyneux, David M. Goldfarb, Kondwani Kawaza, Alinane Linda Nyondo-Mipando

**Affiliations:** 1 Department of Pediatrics and Child Health, College of Medicine, University of Malawi, Blantyre, Malawi; 2 Department of Obstetrics and Gynaecology, BC Children’s and Women’s Hospital and University of British Columbia, Vancouver, Canada; 3 Queen Elizabeth Central Hospital, Pediatrics, Blantyre, Malawi; 4 Department of Pathology and Laboratory Medicine, BC Children’s and Women’s Hospital and University of British Columbia, Vancouver, Canada; 5 School of Public Health and Family Medicine, Department of Health Systems and Policy, College of Medicine, University of Malawi, Blantyre, Malawi

**Keywords:** Bubble CPAP, Neonates, Caregiver, Perspectives, Peers, Malawi

## Abstract

**Background:**

Severe respiratory distress is a leading cause of mortality among neonates in Malawi. Despite evidence on the safety, cost effectiveness and efficacy of bubble continuous positive airway pressure (CPAP) in managing the condition, its use in Malawian health facilities is limited and little is known about caregivers’ engagement with perspectives of bubble CPAP. The purpose of this study was to explore caregiver perspectives for bubble CPAP at both central and district hospitals and key factors that enable effective caregiver engagement in Malawi.

**Methods:**

This was a descriptive qualitative study employing secondary analysis of 46 health care worker in-depth interviews. We interviewed the health workers about their thoughts on caregiver perspectives regarding use of bubble CPAP. We implemented the study at a tertiary facility and three district hospitals in southern Malawi. This was a part of a larger study to understand barriers and facilitators to implementing neonatal innovations in resource-constrained hospitals. Interviews were thematically analysed in NVivo 12 software (QSR International, Melbourne, Australia). Health workers were purposively selected to include nurses, clinicians and district health management involved in the use of bubble CPAP.

**Results:**

Emerging issues included caregiver fears around bubble CPAP equipment as potentially harmful to their new-borns and how inadequate information provided to caregivers exacerbated knowledge gaps and was associated with refusal of care. However, good communication between health care providers and caregivers was associated with acceptance of care. Caregivers’ decision-making was influenced by relatives and peer advocates were helpful in supporting caregivers and alleviating fears or misconceptions about bubble CPAP.

**Conclusions:**

Since caregivers turn to relatives and peers for support, there is need to ensure that both relatives and peers are counselled on bubble CPAP for improved understanding and uptake. Health workers need to provide simplified, accurate, up-to-date information on the intervention as per caregivers’ level of understanding. Notably, contextualised comprehensible information will help alleviate caregivers’ fear and anxieties about bubble CPAP.

## Background

Though nearly all neonatal deaths are preventable [1], there are still 3,600,000 newborn deaths a year worldwide, 99% of which occur in low- and middle-income settings [2]. Sub-Saharan Africa (SSA) alone contributes 39% of all global neonatal death and includes eight of the ten countries with the worst indicators globally [3, 4]. The goal towards elimination of preventable infant mortality is reaffirmed in the Sustainable Development Goal 3.2 and aims at reducing neonatal mortality to 12 per 1000 live births by 2030 [5].

Although Malawi achieved the Millennium Development Goal for reducing mortality rates of children under five by 73% between 1990 and 2015, progress on reducing neonatal deaths has been much slower [6]. By 2015, the main causes of neonatal deaths in the country were prematurity (33%), birth asphyxia and trauma (25.8%) and sepsis (18.6%) [4]. At 18.1%, Malawi registers as one of the highest preterm birth rates globally [7].

The nasal bubble continuous positive airway pressure (CPAP) is a respiratory support strategy that has been successfully utilized for preterm, low-birth weight infants with respiratory failure [8–10], and a low-cost bubble CPAP system was developed in Malawi [10, 11]. However, despite demonstrating cost-effectiveness as well as feasible in improving the survival of children with respiratory distress [12], its use in Malawian facilities is limited. A study done at Queen Elizabeth Central Hospital (QECH) on caregivers’ perception of bubble CPAP indicated that caregivers had little or no information about the intervention and often had negative perceptions about CPAP, which was associated with refusal to accept services [13]. Our objective is to describe and contextualize caregiver perspectives for bubble CPAP in Malawi at both tertiary and district level and explore factors that enable effective caregiver engagement.

For operational purposes, we defined caregiver as mother or any legally accepted unpaid person responsible for giving care to a neonate on bubble CPAP. We excluded relatives unless they were on the bedside as guardians to the neonate and could provide consent on behalf of the legal guardian. We excluded cases where the legal guardian was unavailable due to ill health.

## Methods

### Study type

This is a secondary analysis of 46 semi-structured, in-depth interviews with health workers in southern Malawi on their experiences of using bubble CPAP as part of a larger project, “Integrating a neonatal healthcare package for Malawi” which seeks to inform the scale-up of low-cost and locally appropriate innovations to improve newborn care at low-resource health facilities. The project is a part of the Innovating for Maternal and Child Health in Africa (IMCHA) initiative funded by the Canadian International Development Research Centre (IDRC), Global Affairs Canada (GAC) and the Canadian Institutes for Health Research (CIHR). Through health worker interviews, we sought to understand caregivers’ perspectives on their experiences on using bubble CPAP.

### Study setting

The study was conducted at a tertiary hospital and three secondary level hospitals (hereafter referred to as district 1, district 2 and district 3) in the southern region of Malawi. The tertiary and districts 1 and 3 are public government facilities and provide services for free while district 2 is a private mission hospital. While the mission hospital charges a fee for its services, it serves as a referral centre for the district and clients referred from public facilities access care for free. The tertiary facility administers bubble CPAP in the nursery unit and paediatric nursery ward. The district facilities all provide bubble CPAP in smaller rooms within the nursery units. In total, there are two tertiary and 11 district facilities in southern Malawi. The facilities were chosen in consultation with Ministry of Health. More information is provided a facility assessment paper (see Kawaza et al., 2020 [14] and a primary paper on bubble CPAP (see Nyondo- Mipando et al., 2020 [15].

### Recruitment and selection

The sample was purposively drawn to include health workers involved in delivery and/or decision-making for newborn care at the four health facilities. At the tertiary facility, we recruited nurses and clinicians working in neonatal units, nurses in charge of the ward, registrars and pediatric consultants while at district facility, we recruited nurses and clinicians engaged in neonatal units plus district health officers (DHOs), district medical officers (DMOs), district nursing officers (DNOs), nurses in charge of the pediatric ward. We approached health workers in person and or by phone and asked for an interview after briefing them on the study. Based on the number of health workers that interfaced with bubble CPAP and the limited number of staff available for neonatal care especially at district hospitals, a sample size of 10–15 participants was estimated at each site as being needed to achieve data saturation with a variety of perspectives.

### Interviews

We used in semi-structured interview guide for its ability to allow participants to detail their personal experiences. A team of five trained data collectors conducted 46 interviews with health care workers on perspectives of caregivers towards bubble CPAP. Data collection took place between June and August 2018 (for more information, see Nyondo- Mipando et al., 2020 [15]). We piloted the interview guide and made corrections to the tools before data collection commenced. Data from the pilot phase was not included in the final analysis. Face-to-face interviews were conducted in a secluded place within the facilities and lasted 30–60 min each. Participants provided written informed consent and filled out a demographics form. Interview questions focussed on training, initiation, monitoring, differences in opinions, perception and personal experiences and perception on caregiver understanding of bubble CPAP.

### Data analysis

Data was analyzed using an iterative thematic approach where the research team first familiarized themselves with the interviews to develop a coding framework to be analyzed using NVivo 12 software (QSR International, Melbourne, Australia). The iteration process was applied as developed by Srivastava, 2009 [16] while the thematic analysis was applied as developed by Braun and Clark, 2006 [17]. Analysis commenced during data collection period and involved a constant loop process with researchers reflectively referring back to raw data. SS and ALNM listened to the audios in both languages (Chichewa and English) while MLWK reviewed the transcripts after translation to English. All three read the transcripts several times to familiarize with the data and then generated initial codes together. Where differences arose, all three researchers discussed the suggested codes, constantly referring bact to the raw data until a consensus was reached. The initial transcript was coded by SS and MLWK and codes were compared and areas that differed were discussed between the two researchers until a consensus was reached regarding the most plausible code for that aspect. Thereafter, ALNM checked the transcipt and if any differences arose, all three researchers constantly referred back to the raw data. After completion of each transcript coding, all three researchers referred the coded transcript back to the raw data, objectively reflecting if the codes sppoke to the data. We searched for themes from the codes by organising all similar and recurrent. We examined each code for further subcategories [17]. We reviewed the themes and this resulted in maintaining, combining, separating or discarding themes as necessary [17] and the decision to change was dependent on the richness and breadth of the proposed theme to accommodate other sub themes. For instance, we combined findings on support received from significant others into one theme. We did this to ensure that all themes presented had rich data that substantiated the theme, which resulted into discarding of themes without supporting data. Before discarding, SS and MWLK also reflectively referred back to the raw data to verify if we were making the right decision. We refined the themes and verified our results against the digital recordings. The refined themes were checked for quality by ALNM.

## Results

### Participant characteristics

We conducted interviews with 46 participants and of these, 27 were females. The median age was 32.5 years [IQR 27.3, 39]. One participant dropped out during an interview due to time constraints. Thirty of the 46 participants were nurses. The median length of service as a health care worker was eight years [IQR 3,15] while the median number of years of experience in using bubble CPAP was three [IQR 1.2,4]. (For detailed description, see Nyondo-Mipando et al., 2020 [15]).

### Brief description of the wards

District level hospitals have Neonatal Care Units staffed by clinicians (medical officers or clinical officers) and nursing staff (registered nurse midwives or nurse midwife technicians). The tertiary hospital has a Neonatal Care Unit as well as several High Dependency Units (HDU) and wards where bubble CPAP is administered to infants and children. These units are staffed by pediatricians, registrars, medical interns, registered nurse midwives and nurse midwife technicians. For more detailed description, refer to facility assessment, Kawaza et al. 2020 [14].

### Caregivers’ perspectives on bubble CPAP

Three broad themes emerged from discussions with health care worker about caregiver perspectives on bubble CPAP: fear of bubble CPAP equipment, communication with caregivers and the need to extend counselling beyond the mother.

### Fears about bubble CPAP

According to health workers interviewed, the caregivers’ perspective was primarily that of fear. This fear was included fear of oxygen and its relationship to the severity of illness (and death), fear of pain induced by the tubes, fear of the water in the machine and fear of coffin-shaped baby cots.

### Fear of oxygen and its relationship to severity of illness (and death)

Participants indicated that caregivers were more familiar with the use of therapeutic oxygen and often referred to bubble CPAP as oxygen and transferred their misconceptions from oxygen therapy to bubble CPAP. There was a belief that the provision of oxygen related to the severity of illness and death. Instead of being therapeutic, oxygen was seen to hasten death of the patient so the health facility and providers did not have to provide further care. This can be seen from a quote of a district health officer,

*“There is misconception in the villages that oxygen kills people so women when they see you doing that they think we are trying to kill the child … They feel that it will just kill the child and do not understand that you are trying to help … they didn’t think that you [had] the best interest in your heart for the child. They thought that you just want to finish it.”* District health officer

*“Most of them need psychological support since they fear the machine, mixing up with misconceptions from the village that even oxygen can kill a human. So, we reassure them that it is completely safe to use the machine and that the baby will get better.”* District health nurse

Parental fears were especially strong if caregivers had previously seen or heard of a baby or elderly person who died while receiving oxygen.

*“The common perceptions is that putting a baby on O*_*2*_*(oxygen), s/he will die because it means the baby is seriously ill … or that oxygen kills because most of the times when you put patients on 0*_*2*_*, when they are very, very sick and … most of them...don’t turn do well. So most people think that’s the reason … , that [it] is causing the deaths. … We try to convince her (the mother), [but] … the mothers of those babies who had died on O*_*2*_*, even when they were mourning, they were mentioning something like the O*_*2*_*… [and] the tube thing is what killed the babies.”* Tertiary facility nurse

These associations between bubble CPAP and death are further compounded by the lack of adequate supervision provided by health workers due to overcrowding and understaffing of facilities,

*“ … the same person works in the nursery ward … in maternity. If he works in labour ward, he works in post-natal and KMC. So most of the times, maybe we miss or maybe we fail to check at that recommended hour because may be at that moment you have got a delivery or something in labour ward.”* District facility nurse

### Fear of pain due to equipment tubing

Caregivers were also fearful of the tubes and were worried that the nasal tubes interfered with the baby’s ability to breathe. As one nurse from the central hospital said,

*“The first thing is that they are afraid of that machine, just by looking at it, it actually frightens them … . They always start to think of those tubes entering inside the nostrils of their babies. They think that the tubes will actually block the air to pass through the nose of that baby and … making the child not to be able to suck properly.”* Tertiary facility nurse

The fear of nasal prongs was so strong that some mothers tried to intervene by removing them when the nurse was not watching. To them, removing the nasal prongs offers the baby relief.

*“[W] hen we leave the ward … some will remove the nasal prongs. They put somewhere [and] when they heard your footsteps that we were coming, they tried to pretend to take it to put there and they will tell you, “ah the baby has removed it”. So sometimes it (CPAP) can be seen as … not working … Behind doors, they are removing the baby, stealing some minutes without giving the pressure, the oxygen.”* Tertiary facility nurse

A nurse recalled her first impressions of bubble CPAP when she transferred to the central hospital. Her own reflections were helpful for her to relate to caregivers who may encounter bubble CPAP for the first time when it is used with their sick newborn. In her words,

*“I tensed up when I saw so many machines connected to a small child. I would ask myself so “what is this?” until the bubble CPAP nurse explained … that it is used to supply oxygen to the babies that are getting less oxygen on their own and whose alveolar has collapsed … . It still took me some time to understand … There were so many tubes and connection … it was completely new to me; even on mothers, it is difficult for them to understand. … Looking back to my personal experience, I think the mothers need proper explanation … I was so afraid when I saw the baby on bubble CPAP for the first time; imagine then for someone who is non-medical? She has never seen this, her newly born baby is connected the tubings … , what about the mother of the baby whom she should have been breastfeeding but only to find her baby connected to tubes with the whole face covered … ”* District facility nurse

### Fear of the water in the machine

The bubbling sounds of the bubble CPAP machine were also feared by caregivers. Similar to fears that the tubes blocked the passage of air, caregivers were scared that the baby was inhaling the water.

*"They say [that] the child dies because of inhaling that water … because after bubbling, the water level will [be lower and we have to] add more water, so because of that they think the waters are going down and block the child"* Tertiary facility nurse

*“ … a certain mother consented that the baby should be on CPAP but later on when she saw that it’s a machine which has like boiling water inside, she got terrified and declined to have the baby on CPAP … So seeing such a big machine producing that kind of sound and the water there, to someone who does not understand, it is not easy. It can cause anxiety”* District facility nurse

### Fear of coffin-shaped baby cots

According to health care providers, caregivers’ fears of the association of the treatment with death were additionally fuelled by the shape of baby cots in the nursery rooms. Caregivers associated the cots as baby coffins. One nurse expressed these sentiments:

*“When you look into the nursery, there are cubicles … They are afraid of them, they say that they are coffins. When they were just brought in all the people who were there left saying we have brought coffins. They literally escaped. We failed even to explain to them. They wouldn’t understand and left. It took extra effort to continue explaining when admitting the babies that there are such cubicles in the ward and that they are just ordinary beds for children and not coffins.”* District facility nurse

*“ … especially our nursery, it has boxes which look like the box which we provide the cots, baby cots. So here they believe that those are baby coffins, when you put a baby in there they die. So you have to explain to them.”* Tertiary Facility Nurse

For understanding purposes, these cots are sometimes available even in normal wards (though rare). In most cases, because of shortage of resources, a baby in a critical condition will be prioritised and be given a cot while other babies will sleep on the mother’s beds. It would be safe to assume that most caregivers have seen the cots at one point while visiting the hospital in the children’s, post-natal or female ward. Caregivers might possibly relate graveness of baby’s condition to fear of death, hence the fear that the cots look like coffins.

The fears were a full sensory experience that included both the sounds as well as the appearances of the machines. As the quotes of health care workers reveal, there was a need to explain the system to caretakers in order to help alleviate some of their fears, which is discussed further in the next section.

### Communication with caregivers

Health professionals often cited poor communication in cases where mothers were resistant in accepting bubble CPAP, this was thought to be due to a lack of understanding particularly when health care workers were unable to offer detailed information. First time and young mothers were particularly hesitant. As a district medical officer shared,

*“You understand that sometimes with our numbers that we have in the ward, detailed explanations might not always be available or made available to parents. So sometimes, they have little information and sometimes they come to the hospital with some myths or assumptions, what they have heard before coming to the hospital. ”* District health officer

Inadequate communication is sometimes a result of staff shortages or heavy workload as highlighted in the case below:

*“I’m not going to say we offer the best explanation, but you understand that sometimes with the numbers in the ward, detailed explanations might not always be available or made available to parents. So sometimes they have little information and sometimes they come to the hospital with myths or assumptions, sometimes they are just overwhelmed to see the baby so sick and they are not in a position to understand something very new to their baby, maybe the baby might have been very sick, malaria, LA, Artesonate, IV Fluid, it might be overwhelming and have misconceptions, myths or little information.”* District facility nurse

A case below illustrates a case of poor information due to health workers assumptions that the guardian knew or understood CPAP,

*“ … most of health workers have that perception that if they understand something in the hospital then the mother should obviously also understand … . And because that perception … we don’t take much time explaining to the mother because we assume that they already understand just because we understand, we know the importance, we just explain in a short way without telling them as a mother who doesn’t know anything about the machine you want to put their baby on.”* District health officer

A number of health professionals highlighted the importance of good communication that was empathetic and respectful with the focus on building trust and understanding the anxieties a mother may have with their sick newborn. For example, a clinical officer from a district hospital said,

*“In Malawi we have to acknowledge people think when you initiate a baby on CPAP, it will kill the baby. So we need to explain well to the mom and explain the condition that he [the baby] … has RDS (respiratory distress syndrome). His lungs are like this. So this machine helps in A, B, C. Don’t rush the process. Those women have hope in us and if you explain properly, they understand.”* District clinical officer

A district decision maker further emphasized the importance of a genuine explanation,

*“So number one is your relationship with the guardian. Because that relationship will also go together with you explaining everything that you want to do so that the guardian can understand. Again, what can make them not accept is how you explain … if the explanation is not enough, I think [that] even me, I wouldn’t accept.”* District medical officer

A quote from the nurse emphasizes the importance of good communication:

*“Those women have hope in us and if you explain properly, they understand.”* District facility nurse

A good example of explaining bubble CPAP to a mother can be illustrated in the excerpt below from a nurse at a district hospital. He used gloves to make a visual demonstration of the process of bubble CPAP with the baby’s lungs, which increased the caregivers’ understanding and enabled them to make an informed decision. He said that caregivers typically understood its benefit and gave consent,

*“I put the nasal tubings in the gloves and tighten very well as if you are inflating a balloon and then the glove inflates. I tell the mother to relate how the glove has inflated to as what would happen to the lungs of the child such that the baby will no longer have flat lungs but they will be inflated such that suffocation is reduced and most parents honestly agree, I have never seen a parent who denied the machine, most parents realise fast”* District facility nurse

### A need to extend counselling beyond the mother

The role of significant others in decision-making was also notably highlighted. A nurse from a district hospital below stressed that while counselling and consent have focused on the mother of the infant, there is a need to consider the influence of relatives in the Malawian context.

*“There was a baby who was on CPAP but due to influence of other people who just come to visit the patient from the village, upon seeing the machine, they start discouraging the mother. And if a baby has died within the ward, another baby, with a different condition, they take that incident as a reference to discourage the mother.”* District facility nurse

*“We counsel them about their child or baby or neonate that they can benefit from CPAP machine … but now you find they say “let’s consult our relatives let’s call.”* District clinical officer

Decision-making was described as a collective process involving relatives such as husbands and mother-in-laws. The importance of extending counselling beyond the mother was especially true for young mothers. A nurse from a district hospital elaborated,

*“They also depend on their mothers to help them in making decisions … it was the mother actually who was … trying to convince the owner of the baby for the baby to be removed from CPAP … [S] he reached the point that she called the husband at home and the husband [and] even her father came and when they came they were already informed that the baby was on tubes and they were so furious … In the end … we had to remove the baby on CPAP”* District facility nurse

*“ … these young ones can’t even make decisions on their own. They rely on those who have come with them for decision making.”* District facility nurse

While it was sometimes difficult for nurses and clinicians to adequately explain bubble CPAP to caregivers in a way they understood, health workers interviewed emphasized how peer advocates helped in alleviating fears among caregivers. Encouragement, support and reassurance received from fellow caregivers played a significant role towards helping incoming caregivers to accept assistance. A nurse at the central hospital shared a story of a mother with nine children who initially refused,

*“We initiated and the baby improved. We told her look at this and tell your friends how the machine works to your friends. She started telling them right there in the hospital. Another woman also came and this woman told her that I was also going to lose my child because of such issues but look at her now she has improved. So the other mother also listened to her and also initiated her child.”* District facility nurse

## Discussion

We interviewed health workers to learn caregiver perspectives on bubble CPAP and explore strategies to improve acceptance. Emerging caregiver perspectives about bubble CPAP included fear arising from misconceptions about oxygen therapy, the tubes attached to the baby’s nose, coffin-shaped baby cots and bubbling sounds made by the device. Our findings on parental fears related to the bubble CPAP machinery (bubbling water and tubes) add to global findings that also concluded that tubes, and coffin shaped beds caused anxiety and discomfort among mothers [13, 18, 19].

This study serves to emphasise similar findings that have explored on the negative consequences of insufficient training and experience, sporadic information given to caregivers and shortages of staff as barriers to uptake [13, 20]. Inadequate information dissemination has also been reported in low-resource settings particularly during night and weekend shifts when there is the least staffing [21]. Inability to adequately engage caregivers in decision making [22] compounded by high illiteracy rates [23] are reported as factors affecting caregiver perspectives on health services. This paper highlights the need for enabling health-systems to adequately engage caregivers with new technologies for sick neonates, particularly at tertiary and district hospital settings.

Our study reported that caregivers were afraid of oxygen as they associated it with death. This finding remains consistent with a study by Gondwe et.al [13]. in which misconceptions about bubble CPAP and, oxygen and perceived seriousness of illness largely influenced parental fears [13]. We argue that negative perceptions of therapeutic oxygen may also be in part contextual due to experiences in Malawi in the pre- antiretroviral (ARV) treatments era when mortality due to HIV/AIDs epidemic was high [24]. Paediatric incidence rates of death during the Pre-ARTs period in Malawi due to HIV associated illnesses such as pneumonia and tuberculosis were 191 per 100,000 population in 2011 for tuberculosis (TB) and 11% in 2009 for HIV [25]. However, like Gondwe et al. [13], we also report a shift in opinions among mothers and other caregivers after noticing positive outcomes in babies initiated on bubble CPAP.

The current study indicated that decision making in Malawi is a shared responsibility with family and trusted friends. This necessitates the involvement of significant others like parents-in-law and husbands during information and counselling sessions for informed decision-making. This is in line with other studies that report on addressing fear, anxiety, anger [26], guilt [27], worry and lived experiences [28] between caregivers and their significant others for informed shared decision-making.

Peer support provided through lived experiences by fellow caregivers was perceived to have a positive influence towards alleviating fears among caregivers through encouragement, support and reassurance. Considering staff limitations in the local settings, there is potential to further develop peer support to improve engagement with caregivers. The role of mothers and other caregivers in filling in this aspect has already been explored in low-resource settings [13, 29], such as among neonatal intensive care units and premature babies in Iran [27, 28], however, no study has been conducted specifically looking at peer support among caregivers with babies on bubble CPAP.

Task-shifting of counselling to patient attendants may be an area worth exploring in Malawi that is more sustainable than placing this expectation on caregivers. Patient attendants are non-clinical staff employed to undertake routine duties. With training and potentially a checklist of issues to counsel on, patient attendants could play a vital role in teaching and supporting parents on how the machine works and how to perform routine tasks like holding, feeding and changing nappies while on CPAP as well as providing psychological support to caregivers. Other studies have reported on caregivers’ discomfort with providing such routine tasks to babies and such support will reassure caregivers and calm their fears [13, 19].

A strength of the study is that it combines tertiary and district facilities which broadens the scope of our findings. A limitation of the study is that we interviewed health workers as secondary sources of caregiver perspectives. Future studies should conduct interviews directly with the caregivers themselves. Another limitation is that the study used qualitative methods and therefore findings are not transferable to other settings. However, it does serve to understand practices in other settings.

A unique contribution in our study is the effectiveness of a simple and low-cost effective innovativeness in our local settings that can be applied to aid informed decision making among caregivers using locally found materials. A case of a water-filled balloon or glove serves as a practical example. Considering the limitation of resources in low income countries, such an approach would be a novel idea.

## Conclusion

This study highlights a number of potential, low-cost solutions that can be employed to improve the acceptance of bubble CPAP among caregivers in Malawi. This includes simplified and contextualised decision aides and extending counselling beyond mothers.

## Data Availability

The datasets used and/or analysed during the current study are available from the corresponding author (SS) on request.

## Abbreviations

ART: Antiretroviral Treatment
AIDS: Acquired Immunodeficiency Syndrome
CIHR: Canadian Institutes for Health Research
CPAP: Bubble Continuous Positive Airway Pressure
DHO: District Health Officers
DMO: District Medical Officers
DNO: District Nursing Officers
GAC: Global Affairs Canada
NAC: National AIDS Commission
IDRC: Canadian International Development Research Centre
IMCHA: Innovating for Maternal and Child Health in Africa
QECH: Queen Elizabeth Central Hospital
RDS: Respiratory Distress Syndrome
SSA: Sub-Saharan Africa
TB: Tuberculosis
